# Green technology innovation and carbon emissions nexus in China: Does industrial structure upgrading matter?

**DOI:** 10.3389/fpsyg.2022.951172

**Published:** 2022-07-26

**Authors:** Pengfei Gao, Yadong Wang, Yi Zou, Xufeng Su, Xinghui Che, Xiaodong Yang

**Affiliations:** ^1^School of Marxism, Xinjiang University, Ürümqi, China; ^2^School of Marxism, Liaoning Petrochemical University, Fushun, China; ^3^School of Tourism & Transportation Management, Guang Xi Eco-Engineering Vocational & Technical College, Liuzhou, China; ^4^School of Economics and Management, Xinjiang University, Ürümqi, China; ^5^School of Economics and Management, Tarim University, Alar, China; ^6^School of Public Administration, Liaoning University, Shenyang, China; ^7^Center for Innovation Management Research of Xinjiang, Xinjiang University, Ürümqi, China

**Keywords:** green technology innovation, industrial structure upgrading, carbon emission reduction, mediating effect, China

## Abstract

Compared with traditional technological innovation modes, green technology innovation is more targeted for low carbon development and critical support for countries worldwide to combat climate change. The impact of green technology innovation on carbon emissions is considered in terms of fixed effect and mediating effect models through industrial structure upgrading. For this purpose, the sample dataset of 30 provincial administrative areas in China from 2008 to 2020 is employed. The results demonstrate that green technology innovation exerts significantly inhibitory effects on carbon emissions, whose conclusion still holds after removing municipalities and replacing the dependent variable. Industrial structure upgrading is vital for green technology innovation to diminish carbon emissions. There is significant regional heterogeneity in the effects of green technology innovation on carbon emissions, i.e., the direct and indirect impact of green technology innovation on carbon emission reduction is significant in the eastern-central area, but its effect is insignificant in the western region. Therefore, it is essential to realize carbon emission reduction by further bolstering green technology innovation and accelerating industrial structure upgrading to fulfill the synergy of technology and structure.

## Introduction

Since 1978, China has produced extraordinary gains in economic construction that have placed it among the second largest economies in the world (Wei et al., 2017). Nevertheless, underlying the high-speed growth, its environmental carrying capacity is increasingly strained (Abbasi et al., 2022). Moreover, a development model that ignores environmental considerations by depending on high inputs and the high energy consumption is intolerable, with carbon emissions in China close to 30% of the global carbon emissions (Li et al., 2022; Zhao S. et al., 2022; Zhao W. et al., 2022). The climate challenges induced by high energy consumption, high pollution, and increased carbon emissions are not only limited to local development challenges but also the cross-border nature of carbon emissions, which constitute a joint problem worldwide at present (Godil et al., 2021; Huo et al., 2022; Rehman et al., 2022). Therefore, governments are paying unprecedented attention to this matter and adopting aggressive carbon emission reduction policies to combat global climate change (Amin et al., 2020; Gyamfi et al., 2022; Liu et al., 2022). Under the double burden and eagerness for international and domestic carbon emission reduction, President Xi Jinping suggests that “the Chinese government will raise its contribution and introduce a more practical approach in striving to peak carbon emissions by 2030 as well as realize carbon neutrality by 2060” (Fang et al., 2019; Sun et al., 2020; Jia et al., 2022).

Moreover, because of institutional constraints and economic development processes, the Chinese government mainly emphasizes conventional technology breakthroughs but seldom devotes itself to green technology, thus lacking technology factors in this field. The double carbon goal aims to shift the economy toward the green and low-carbon stage, while green technology innovation is considered a key ingredient to driving such transformation. To further pursue green transformation, the Chinese government has explicitly introduced green technology innovation from the technology segment based on exploring carbon peaking and carbon neutrality (Ren et al., 2021; Shi and Xu, 2022; Shi et al., 2022). The Chinese government also notes that as low-carbon economic system is reformed and developed, green technology innovation is emerging as an influential driving force. Some scholars state that compared with defensive and adaptive ones, such as eliminating high-carbon production capacity and reducing emissions at the source end, green technology innovation is not only a chain-wide transformation but also can reduce the premium price of green products and reduce the cost of combating climate change (Chien et al., 2021; Suki et al., 2022; Wang et al., 2022). It is, however, not well covered by past studies whether there is an impact of green technology innovation on carbon emissions. Green technology innovation and carbon emission serve as significant thrusts and objective functions for pursuing the double carbon goal, respectively, which may have a nonnegligible intrinsic correlation (Dong et al., 2022). Therefore, against this background, it is helpful to investigate and exert the effect of green technology innovation on carbon emissions to correctly grasp the future development direction of technology innovation and industrial transformation and upgrading as well as the low-carbon policy system.

It is noteworthy that, as part of the core content of the supply-side structural reform, the industrial structure is intrinsically associated with carbon emissions (Torvanger, 1991; Guo et al., 2021). Until 2020, the share of the output value of three industries in China is 7.7, 37.8, and 54.5%, with the industrial structure advancing toward an advanced level and realizing the development mode of mainly secondary and tertiary industries.^1^ Industrial structure upgrading contributes to economic development direction and low carbon governance, a significant channel influencing carbon emissions (Dong et al., 2020). Simultaneously, green technology innovation will be directly applied to various industries, its role and application in different industries are various, and its effects on carbon emissions in each industry are also different. So, this article wonders how do green technology innovations affect carbon emissions? What are the heterogeneous characteristics of the effects of green technology innovation on carbon emissions in different geographical locations? How does the role of green technology innovation on carbon emissions in the context of industrial structure upgrading? The previous literature is devoid of a discussion on these subjects. Therefore, it is essential to examine the impact of green technology innovation and industrial structure upgrading on carbon emissions, which can not only contribute to the decision-making reference for the Chinese government to formulate and optimize low-carbon development policies but also serve as a reference for the low-carbon path of related emerging economies.

Compared with the available literature, this article's marginal contribution is probably in three aspects. First, unlike technological innovation, incorporating the more targeted green technological innovation and carbon emission into one coherent research framework to bridge the gap between green technological innovation and carbon emission inhibition. Second, this article elaborates on the influences of green technology innovation on carbon emission in the light of industrial structure upgrading, which not only aims to effectively capture the evolutionary channels of green technology innovation and industrial transition and upgrading but also benefits building a scientific and sound carbon emission reduction policy framework. Finally, the survey sample is grouped into the eastern-central and western areas to evaluate the relationship between the three, which forms the basis for tailoring the low-carbon development system to local conditions.

The remainder of the text is structured as follows. A literature review that organizes and reviews the impact of green technology innovation and industrial structure upgrading on carbon emissions is discussed, followed by the research design that includes detailed methodology, variable definitions, and data sources. The empirical results are described in detail and a comparative discussion is provided. Finally, the article includes research conclusions, policy implications, deficiencies, and future research directions.

## Literature review

Carbon emissions are oriented to the ecological environment and the science and technology innovation industry. In contrast, science and technology have emerged as a lever to advance industrial structure change, which means that “the scientific revolution and the technological revolution spearheaded by the scientific revolution are surging forward, with the technological revolution transforming modern production and modern life in an unprecedented manner” (Rip and Kemp, 1998; Chen, 2022; Hu et al., 2022). Scholars have scrutinized carbon emissions from various viewpoints, especially technological innovation, which has gained much attention as a critical contributor to combating climate change (Wang et al., 2019; Bai et al., 2020; Khan et al., 2020). Judging from available literature findings, it is generally believed that the impact of technological innovation on regional carbon emissions usually has a dual effect. On the one hand, technology innovation reduces carbon emissions derived from efficiency gains in using energy factors, cost savings, and various spillover effects of technology innovation (Feng et al., 2020; Rehman et al., 2021). For example, Karen et al. (2006) found that corporate human capital reinforces innovation and reduces energy intensity, thereby controlling carbon emission levels. Meirun et al. (2021) affirmed that in Singapore, green technology innovation curbs carbon emissions both in the short and long term.

Moreover, Soares and Tolmasquim (2000) and Siitonen et al. (2010) demonstrated that both energy efficiency and energy mix improvements effectively reduce CO_2_ emissions. Wang et al. (2013) examined the influencing factors of carbon emissions in Guangdong Province utilizing a ridge regression to fit an extended STIRPAT model; all conclude that technological innovation may enable carbon emission reduction. Xu and Lin (2016) explained that significant heterogeneity is observed in terms of the directionality of technological progress in mitigating carbon emissions. On the other hand, technology innovation may also have a facilitative effect on carbon emissions, i.e., along with the concentration of economic activities, an increase in technology innovation will trigger higher carbon emissions. Such research emphasizes the negative externalities of technology innovation on the ecological environment, which numerous scholars support (Van den Bergh, 2013; Ali et al., 2020; Sarfraz et al., 2021). Berkhout et al. (2000), for example, argued that the energy “rebound effect drives the carbon emission growth”. Although technology innovation can conserve resources by boosting energy efficiency, production costs and energy prices also decrease with technological advancement, which, in turn, broadens energy demand and consumption, and eventually, the two offset each other (Ottman et al., 2006; Yasmeen et al., 2022). Jaffe et al. (2002) argued that technological progress has a complex impact on carbon dioxide emissions, with uncertainty effects that do not exactly contribute to the reduction effect. Weina et al. (2016), using data from Italy in their study, revealed that green technology innovation significantly enlarges environmental productivity but does not considerably better environmental quality and that this difference is not motivated by regional differences. Wei and Yang (2010) argued that the ultimate effect of technological innovation on carbon emissions lies in a double-effect wrestle, where the net result is demonstrated as a carbon emission reduction. Conversely, a carbon emission promotion occurs when the impact of technological innovation to inhibit carbon emissions is greater than the energy rebound effect. That mere technological innovation enhancement is not able to impede carbon emissions. More carefully, some scholars also suggest that technological innovations, because of their different attributes, embrace those that expand production and those that treat pollution and reduce emissions, and thus their role in carbon emissions is subject to more significant uncertainty (Jaffe et al., 2002; Chen et al., 2019).

Besides, green technological innovation is a technological innovation that adheres to ecological and economic norms, which differs significantly from technology innovation (Xin et al., 2022). Therefore, many scholars have controversial opinions regarding green technology innovation and carbon emissions and have yet to provide consistent findings. Green technology innovation can diminish carbon emissions. Shan et al. (2021) suggested a long-term cointegration of green technology innovation with carbon emissions and that green technology innovation significantly reduces carbon emissions. Using China as a case study for analysis, Zhao et al. (2021) found that the inhibitory effect of technology innovation and financial risk on global carbon emissions is only statistically significant at the 10th quantile. Obobisa et al. (2022) pointed out that green technology innovation significantly influences carbon emissions. Shahbaz et al. (2020) reached a similar view and employed the BARDL model to reveal the nexus between carbon emissions and their determinants. Empirical findings suggest that technology innovation adversely affects carbon emissions. The second argument is that green technology innovation does not inhibit carbon emission reduction but facilitates carbon emissions. Supporter Du et al. (2019) investigated that green technology innovation does not significantly interfere with carbon emissions when income levels are below a critical value. Moreover, Zeng et al. (2019) use epsilon-based measure (EBM) and data envelope analysis (DEA) models to evaluate the carbon emission efficiency and its differences among 30 Chinese provinces, which indicate that industrial structure, technological innovation, and carbon emission efficiency are significantly and positively correlated. With emerging economies, Razzaq et al. (2021) revealed that green technology innovation's role in carbon emissions is evident only at higher quartiles in Brazil, China, India, and Russia. The utility is positive or insignificant at lower quartiles.

Finally, starting from a carbon emissions view, another significant dimension in carbon emission impact mechanism research is whether the environmental Kuznets curve (EKC) holds. However, considerable empirical evidence is found for the general applicability and functional form of the EKC curve (Shaheen et al., 2022). Bertinelli and Strobl (2005) and Bagliani et al. (2008) empirically study data from developed countries from different perspectives and reject the EKC hypothesis, arguing that insignificant nexus is found between affluence and environmental degradation and that structural change is a determinant of emission reduction. It is speculated that the reason for these inconsistent results is that carbon dioxide emissions have a stronger spatial spillover than other pollution, with its high cost and low benefit resulting in a lack of willingness to actively reduce emissions, as well as being related to the measurement method and sample data selection (Wang et al., 2018; Liang et al., 2019). In addition, omissions and missing variables are reasons for biased estimates, such as energy mix, environmental policies, and industrial structure, which all significantly affect carbon emissions (Stern, 1998; Sharif et al., 2019). Moreover, Zheng et al. (2020) found that variations in local development patterns have contributed to industrial structure upgrading at the regional level to inhibit carbon emissions in most areas. Zhang et al. (2020) developed a comprehensive framework for the impact of industrial structure and technological progress on carbon intensity, suggesting that industrial structure upgrading indirectly increases carbon intensity by promoting technological change.

Judging from reviewing the literature, there is no consensus on investigating the links between green technology innovation and carbon emissions, which is discussed in terms of technological progress and the absence of investigation from an industrial structure upgrading perspective. Green technology innovation tries to reconcile the link between man and nature, that is, to obtain nature's salvation and to achieve the freedom of spirit and the development of man on the premise of grasping the objective laws of nature. Given this, considering 30 provincial administrative areas from 2008 to 2020 as the dataset, this article empirically analyzes the association between green technological innovation and carbon emissions. Furthermore, by employing a fixed-effect and a mediating effect model based on the industrial structure upgrading perspective, this article aimed to enhance transformational green development through technological change further and fulfill “carbon peaking and carbon neutrality” to provide some theoretical reference. In short, green technology innovation seeks to harmonize the relationships between humankind and nature, i.e., to acquire the redemption of nature and to fulfill the freedom of nature and the evolution of human beings while grasping its objective rules.

## Research design

### Model setting

To examine the detailed influence of green technology innovation on carbon emissions, referring to Du et al. (2019) and Bilal et al. (2021), we set up the relevant empirical model in the following equation:


(1) $$\begin{array}{l} {CO_{2}}_{i,t}=\alpha_{0}+\alpha_{1}GTI_{i,t}+\beta W_{i,t}+\varepsilon_{i,t} \end{array}$$

where *i* and *t* characterize province and time, respectively. *GTI* is the core explanatory variable that is characterized by using green technology innovation. *CO*_2_ is the dependent variable that is stabilized using carbon emissions. *W* denotes control variables, which include economic development (*Pgdp*), urbanization (*Urban*), foreign direct investment (*Fdi*), environmental protection (*Ep*), and local fiscal expenditure (*Fe*). ε is the disturbance term.

Following the previous findings, green technology innovation may influence carbon emissions in terms of the industrial structure upgrading toward goals that meet carbon emission reduction. Therefore, a more normative mediating effect model must be constructed for further empirical testing. Borrowing from the stepwise regression mediating effects test that Yang et al. (2021) and Wang et al. (2022) employed, a mediation effect can be considered to exist when two principles are fulfilled in the test process. One is that the basic model's explanatory variable (X) significantly affects the explanatory variable (Y). The second is that every variable included within the dependent link, when its primary variables are manipulated, significantly exerts its influence on subsequent variables. Specifically, the significance of the coefficient of the mediating variable (M) determines whether a mediating effect exist or not. A basic model of the mediation effect test is described as follows:


(2) $$\begin{array}{lll} Y & = & cX+e_{1} \end{array}$$

(3) $$\begin{array}{lll} M & = & aX+e_{2} \end{array}$$

(4) $$\begin{array}{lll} Y & = & c^{,}X+bM+e_{3} \end{array}$$

(5) $$\begin{array}{lll} Upg_{i,t} & = & \alpha_{0}+\rho_{1}GTI_{i,t}+\beta_{1}X_{i,t}+\varepsilon_{i,t} \end{array}$$

(6) $$\begin{array}{lll} {CO_{2}}_{i,t} & = & \alpha_{0}+\lambda Upg_{i,t}+\rho_{2}GTI_{i,t}+\beta_{2}X_{i,t}+\varepsilon_{i,t} \end{array}$$

where *Upg* measures industrial structural upgrading, and the remainder indicators are identical to the above equation. The mediating role of green technology innovation on carbon emissions through the corresponding mediating variables is significant when the coefficients α_1_, ρ_1_, and λ in Equations (1), (5), and (6) are significant. On this basis, it is necessary to investigate whether M in the test model (1) is significant (if it is not significant, revealing that mediating effect is only found, also known as a full mediating effect; otherwise, it is also known as a partial mediating effect).

### Variable definitions

#### Dependent variable

##### Carbon emissions (CO_2_)

No uniform measurement method is available for carbon emission estimation (Deng et al., 2019; Hao et al., 2021). Carbon emissions are considered to be calculated concerning carbon emission factors of eight types of fossil fuels (Table 1) and energy fossil fuel consumption, respectively (Wu et al., 2020; Li et al., 2021). The carbon emission-specific calculation formula is given as follows:


(7) $$\begin{array}{l} CO_{2}=k\cdot \sum_{i=1}^{n}E_{i}\cdot \delta_{i} \end{array}$$

where *CO*_2_ denotes carbon emissions, *k*(*k* = 44/12). *E*_*i*_ is fossil fuel consumption in category *i*. δ_*i*_ is the emission factor for fossil fuel category *i*.

**Table 1:** Carbon emission coefficient of various fossil fuels.

**Fuel types**	**Default carbon content (kgc/GJ)**	**Default carbon oxidation rate**	**Mean low-grade heat generation (KJ/kg, m** ^3^ **)**	**Carbon emission coefficient (kgc/kg, m** ^3^ **)**
Coal	25.8	1	20,908	0.539
Coke	29.2	1	28,435	0.830
Crude oil	20	1	41,816	0.836
Gasoline	18.9	1	43,070	0.814
Kerosene	19.6	1	43,070	0.844
Diesel oil	20.2	1	42,652	0.862
Fuel oil	21.2	1	41,816	0.882
Natural gas	15.3	1	38,931	0.596

#### Core explanatory variable and mediating variables

Green technology innovation (GTI). Green technological innovation is a technological innovation that adheres to ecological and economic norms, aiming at protecting the environment and minimizing the total product cost at each stage of the product life cycle innovation process (Zhang et al., 2022). Compared with a patent granted volume, patent application volume is much more representative of technological innovation results in the application year. It often starts from 1 to 3 years for application to grant, while patent granted is susceptible to uncertainty due to many factors such as testing, annual fee payment, and market environment. Compared with invention patents, design and utility patents have relatively low technology levels that are convenient to learn and imitate, so invention patents can best represent innovation ability. Consequently, depending on the information regarding patenting activities of green technology innovation available from IPC, patent applications for green technology inventions in each area are collected on the patent retrieval and analyzing system of the State Intellectual Property Office. Therefore, this article described green technology innovation characteristics regarding the share of green technology invention in patent applications. Industrial structure upgrading (*Upg*). Industrial structural upgrading is a dynamic process of industrial factor migration from low-productivity to high-productivity sectors. Currently, the dominant measures of the industrial structure are the ratio of value-added of tertiary industries to value-added of secondary industries, the ratio of the sum of value added of secondary and tertiary industries to GDP, or the use of industrial sophistication or rationalization indices to characterize it. In this article, industrial structure upgrading is denoted by a share of tertiary industry in the economy.

#### Control variables

To control for additional interfering factors of the dependent variable, referring to Zhong et al. (2021) and Li et al. (2022); this article introduces control variables, which include economic development level (*Pgdp*), urbanization (*Urban*), foreign direct investment (*Fdi*), environmental protection (*Ep*), and local fiscal expenditure level (*Fe*). Economic development is coupled with deepening industrialization, which is one of the major triggers of carbon emissions. Economic development level (*Pgdp*) is denoted by GDP per capita. Rapid urbanization is driven by energy, and at this stage, China is still dominated by fossil fuels, which, in turn, has an impact on carbon emissions (Wang H. et al., 2021; Wang S. et al., 2021). Urbanization (*Urban*) is measured by the ratio of the urban population to the total rural population. Referring to Ren et al. (2022), foreign direct investment (*Fdi*) is the amount of actual foreign direct investment converted into RMB. The expansion of afforestation can accelerate the absorption of GHGs, which, in turn, has a stabilizing effect on carbon emissions. Following Gao et al. (2014), environmental protection (*Ep*) is measured by the total area of afforestation. If fiscal spending is biased toward low-carbon projects, then it is conducive to reducing carbon emissions, and conversely, it can exacerbate them. The fiscal expenditure level (*Fe*) is characterized by local general budget expenditure.

### Data

This article selects panel data that covers 30 provinces administrative areas in China from 2008 to 2020. The raw data are available from the China Statistical Yearbook, the China Macroeconomic Database, and the EPS Global Statistical Data Analysis Platform for the period under examination. These data are obtained from the State Intellectual Property Office of China regarding green technology innovation. Meanwhile, the International Patent Classification (IPC) is employed to identify the annual number of green patent applications in China. For the missing data in the statistical sources, the neighboring value filling method and the mean value filling method are used to fill them. All variables are logarithmically treated in order to ensure data smoothness. The descriptive statistics on relevant variables are detailed in Table 2.

**Table 2:** Statistical description of variables.

**Variable**	**Obs**	**Mean**	**Std. Dev**.	**Min**	**Max**
CO_2_	390	10.241	0.738	8.045	11.928
GTI	390	−0.791	2.354	−6.479	5.869
Upg	390	3.796	0.202	3.353	4.430
Fdi	390	12.750	1.645	6.167	15.090
Ep	390	11.745	1.331	6.565	13.667
Urban	390	4.018	0.225	3.371	4.495
Fe	390	8.165	0.702	5.783	9.766
Pgdp	390	10.686	0.532	9.085	12.013

## Results and discussion

### Discussion of the baseline regression results

Table 3 reflects the empirical regression results provided by the fixed-effects (FE) model. Meanwhile, to guarantee the scientific rigor of the research results, this article has incorporated the random-effects (RE) and OLS models into the regression analysis to monitor the potential influence of green technology innovation on carbon emissions in the empirical sample. This article demonstrates that green technology innovation significantly stimulates a decrease in carbon emission levels, whether in the fixed-effects model, random-effects, or OLS test. Our results are comparable to studies by Bilal et al. (2021) and Obobisa et al. (2022) but differ from that by Razzaq et al. (2021). Obobisa et al. (2022) pointed out that green technology innovation significantly influences carbon emissions. Green technology innovation can stimulate industries to perform optimization and upgrading by optimizing production processes and refining energy-intensive production patterns, which brings about an elevated production efficiency and thus reduces carbon emissions. As for the green technology innovation, its essence is to minimize the product's total life-cycle cost and carbon emissions by minimizing the environmental cost in all processes, from processing to production. Additionally, because of porter's hypothesis, it can be concluded that a higher green technology innovation level will be bound to produce a “green innovation compensation” effect, which can effectively dampen the negative impact on the environment while improving the industrial competitiveness of enterprises, and finally attaining the goal of carbon emission reduction (Du et al., 2021; Sun et al., 2022).

**Table 3:** Baseline regression results.

**Variables**	**FE**	**RE**	**OLS**
GTI	−0.008^**^	−0.008^**^	−0.041^***^
	(0.004)	(0.004)	(0.015)
Fdi	−0.026^**^	−0.017	0.137^***^
	(0.013)	(0.013)	(0.023)
Ep	−0.040^***^	−0.036^***^	0.127^***^
	(0.014)	(0.014)	(0.028)
Urban	0.357^**^	0.326^**^	0.508
	(0.144)	(0.146)	(0.347)
Pgdp	0.016	−0.050	−0.299^*^
	(0.072)	(0.072)	(0.154)
Fe	0.255^***^	0.307^***^	0.607^***^
	(0.050)	(0.049)	(0.076)
Constant	7.354^***^	7.594^***^	3.173^***^
	(0.437)	(0.446)	(0.882)
Observations	389	389	389
*R* ^2^	0.631		0.529
Number of code	30	30	30

***, **, and * *are significant at the levels of 1, 5, and 10%, respectively, (the same below); Standard errors are in parentheses*.

### Discussion of mediating effect results

The significant role of green technology innovation in carbon emission reduction is identified in this study based on the previous analysis. However, an in-depth evaluation is needed to determine the role mechanism of the two nexus. As mentioned in the previous studies, green technology innovation contributes mainly to carbon emission reduction by acting on industries to achieve structural optimization. Therefore, following the procedure of the specific test for mediating effect described in the previous section to combine Equations (1), (5), and (6) can further confirm the mediating role of industrial structure upgrading between the two (Table 4). Column (1) in Table 4 confirms that the coefficient of green technological innovation is significantly positive, indicating that the increase in green technological innovation level will significantly stimulate industrial structure upgrading at the local level, which is aligned with the findings of Xu et al. (2021). A plausible interpretation is that green technology innovation greatly spurs green demand. Under the role of upstream and downstream effects, the industrial chain can be developed and stretched. Simultaneously, a higher green innovation level can diminish resource energy consumption and production costs, the production factors causing the spontaneous flow to high productivity sectors, and finally driving industrial structure transformation and upgrading.

**Table 4:** Mediating effect results.

**Variables**	**Upg**	**CO**_2_
Upg		−0.369^***^
		(0.084)
GTI	0.015^***^	−0.003
	(0.002)	(0.004)
Fdi	−0.027^***^	−0.036^***^
	(0.008)	(0.013)
Ep	−0.015^*^	−0.046^***^
	(0.008)	(0.013)
Urban	0.844^***^	0.669^***^
	(0.089)	(0.157)
Fe	0.307^***^	0.368^***^
	(0.031)	(0.055)
Pgdp	−0.374^***^	−0.122
	(0.045)	(0.077)
Constant	2.424^***^	8.248^***^
	(0.271)	(0.472)
Observations	389	389
*R* ^2^	0.765	0.650
Number of code	30	30

*** and * *are significant at the levels of 1, 5, and 10%, respectively. Standard errors are in parentheses*.

Moreover, judging from the green product supply side, the emergence and application of green technology innovation hastened the related enterprises to strengthen their speed of building product differentiation barriers and technological advantages, which, in turn, positively impacts the whole industrial structure by catfish effect. Regarding the demand side, compared with traditional products, green technology innovation also drives down their prices. In addition, it stimulates the public's green demand, thus affecting the industrial structure. In conjunction with columns (1) and (2) in Table 4, it is revealed that green technology innovation can serve carbon emission reduction purposes *via* the facilitation of industrial structure upgrading. Against the backdrop of the elevated green technology innovation level, green technology innovation significantly facilitates industrial structure upgrading that can effectively eliminate backward production capacity, improve labor production efficiency, promote industrial development to high value-added and high-tech direction, reduce high energy-consuming and high-polluting industries in economic activities, effectively boost green production benefits, and greatly reduce carbon emissions.

### Discussion of regional heterogeneity results

Because of economic scale and geographical location variations, regional heterogeneity may be observed in the green technology innovation level and industrial structure status. This article analyzes the heterogeneous effects of green technology innovation and industrial structure upgrading on carbon emissions (Table 5). Columns (1) and (4) of Table 5 suggest that the green technology innovation level in the eastern-central areas will significantly stimulate carbon emission reduction by passing the 5% significance level test. However, the carbon emission reduction effect of green technology innovation in the western area is insignificant. The likely explanation is that the relatively scarce human resources, insufficient capital factors, weak technological base, and backward infrastructure in the western area have a practical gap with the eastern and central areas. At the same time, the enhancement of green technology innovation level necessitates a specific necessary foundation. Therefore, compared with the eastern-central area, the realistic regional disparity factor determines that the green technology innovation level in the western area is much lower, which significantly inhibits the carbon emission reduction effect of green technology innovation in the western area.

**Table 5:** Regional heterogeneity results.

**Variables**	**Eastern-central area**			**Western area**		
	**CO**_2_	**Upg**	**CO**_2_	**CO**_2_	**Upg**	**CO**_2_
Upg			−0.415^***^			−0.259^*^
			(0.087)			(0.151)
GTI	−0.009^**^	0.009^***^	−0.004	−0.003	0.018^***^	0.001
	(0.004)	(0.003)	(0.004)	(0.006)	(0.003)	(0.007)
Fdi	−0.070^***^	−0.029^**^	−0.082^***^	0.027	−0.039^***^	0.017
	(0.017)	(0.013)	(0.016)	(0.019)	(0.011)	(0.020)
Ep	0.002	−0.020*^*^	−0.006	−0.068*^*^	−0.015	−0.072*^*^
	(0.013)	(0.010)	(0.012)	(0.032)	(0.018)	(0.032)
Urban	0.610^***^	1.100^***^	1.066^***^	0.126	0.818^***^	0.337
	(0.151)	(0.114)	(0.172)	(0.302)	(0.169)	(0.324)
Fe	0.003	0.235^***^	0.100^*^	0.638^***^	0.296^***^	0.715^***^
	(0.055)	(0.042)	(0.056)	(0.086)	(0.048)	(0.096)
Pgdp	0.260^***^	−0.269^***^	0.149^**^	−0.351^**^	−0.404^***^	−0.456^***^
	(0.072)	(0.055)	(0.073)	(0.136)	(0.077)	(0.149)
Constant	6.069^***^	0.913^**^	6.448^***^	8.589^***^	3.110^***^	9.394^***^
	(0.540)	(0.410)	(0.520)	(0.656)	(0.368)	(0.803)
Observations	234	234	234	155	155	155
*R* ^2^	0.661	0.802	0.694	0.715	0.763	0.721
Number of code	18	18	18	12	12	12

***, **, and * *are significant at the levels of 1, 5, and 10%, respectively. Standard errors are in parentheses*.

Columns (2) and (5) of Table 5 demonstrate that green technology innovation significantly facilitates industrial structure upgrading in both eastern-central and western areas, passing the 1% significance test. The overall and regional samples reveal that the industrial structure upgrading role of green technology innovation is prevalent. Combined with columns (3) and (6) of Table 5, the mediating role of industrial structure upgrading in the eastern-western area indicates significant regional differences. Specifically, in the eastern and central areas, the mediating role of industrial structure upgrading in the carbon emission reduction effect of green technology innovation is partial. In contrast, it presents a fully mediating effect in the western area and passes the 10% significance test. The variability may explain differences in green technology innovation levels, and the variability in industrial structures that also determines their impact on carbon emissions (Du et al., 2021; Razzaq et al., 2021).

### Discussion of robustness results

To confirm that these results are robust, the entire sample is investigated using the following two steps. (1) Two-stage least squares (TSLS) is employed to perform the endogeneity test, where the core explanatory variable lagged one period is used as an instrumental variable. [refer to columns (1) and (2) of Table 6]. (2) The alternative explanatory variables are introduced. The article re-performs the regression validation by replacing the carbon emission variable with SO_2_ emissions [refer to columns (3) and (4) in Table 6]. Table 6 demonstrates that green technology innovation provides a remarkable benefit to carbon emission reduction with a previous robust result.

**Table 6:** Robustness tests.

**Variables**	**(1)**	**(2)**
	**Endogeneity test (TSLS)**	**Replacing the dependent variable**
GTI	−0.257^*^	−0.154^***^
	(0.138)	(0.016)
Fdi	−0.103^***^	0.013
	(0.034)	(0.058)
Ep	−0.116^***^	−0.081
	(0.037)	(0.061)
Urban	0.838^*^	−0.985
	(0.494)	(0.641)
Fe	1.085^***^	−0.792^***^
	(0.306)	(0.224)
Pgdp	−0.188	0.402
	(0.212)	(0.322)
Constant	−2.878	10.310^***^
	(3.774)	(1.949)
Observations	359	389
*R* ^2^	0.257	0.617
Number of code	30	30

***, **, and * *are significant at the levels of 1, 5, and 10%, respectively. Standard errors are in parentheses*.

## Conclusion and policy implications

Since the prevailing economic development situation is characterized by environmental pollution and excessive energy consumption, a green revolution and low-carbon economy have emerged as a shared goal for all countries. It is a simple truth that protecting the ecological environment is to protect productivity, while bettering the ecological environment is to develop productivity. Therefore, how to comply with the general direction of contemporary technological revolution and industrial change, seize the substantial development opportunities brought by green transformation, vigorously enhance the transformation of economic, energy, and industrial structures, and make low-carbon development boost sustainable global economic growth. Utilizing 30 provincial administrative areas in China as the data set from 2008 to 2020, this article employs fixed-effect and mediating-effect models to excavate the impact of green technology innovation on carbon emissions in the light of industrial structure upgrading. Our results prove that green technology innovation significantly mitigates carbon emissions, i.e., as green technology innovation level increases, carbon emission level decreases gradually. Moreover, green technology innovation also indirectly inhibits carbon emissions *via* industrial structure upgrading. Lastly, although the direct and indirect inhibitory effects of green technology innovation on carbon emissions are significant in the eastern-central areas, its effect is not significant in the western area. Therefore, supported by green technological innovation, it is essential for us to vigorously carry out scientific and technological innovation (S&T) innovation to boost a beautiful society with the fundamental goal of ecological and environmental quality improvement and thus aim to develop an excellent natural environment for the comprehensive and accessible development of human beings.

Therefore, some necessary policy actions should be conducted so that carbon emission reduction can be better implemented in conjunction with green technology innovation and industrial structure upgrading.

First, policymakers should capture the linkage between the two in each area and implement differentiated regional policies by combining the fundamental basis of green technology innovation. Policymakers should also adopt appropriate policies on green technology innovation capacity enhancement plans, new energy green industry support, and green technology subsidies and transfer landing application incentives to maximize the role of green technology innovation in carbon emission reduction by formulating corresponding solutions to provide long-term mechanisms to guarantee carbon emission reduction goals.

Second, policymakers should exert the effect of industrial structure on carbon emission reduction. Policymakers can vigorously cultivate new industries motivated by green technology innovation combined with Internet technology and digital technology to facilitate the high-end development of industrial structures to reduce carbon emissions. Policymakers should also strongly support green and clean industries and transform the industrial structure from high emission to high technology and low emission industrial forms. Besides, policymakers should broaden the green technology innovation scope, spur the green demand effect of upstream and downstream related industries, and steer the spontaneous flow of factors to high-productivity sectors to contribute to the green transformation of industrial structure.

Finally, raw Chinese green technology is still weak. Green technology innovation activities involve multiple fields, links, and complex coordination among multiple sectors. Therefore, policymakers should strengthen the top-level system and build a green technology supply system market mechanism oriented to market demand. Meanwhile, each area shall develop a green technology innovation chain with the organic combination of industry, university, and research, the effective connection of all layers of the industrial chain, and synergistic development of large- and medium-sized enterprises to realize a government-led, market-oriented, and synergistic green technology research and development, achievement transformation, demonstration application, and industrialization of the green industry chain. In addition, policymakers should also reinforce green technology innovation investment, fill the short board of green technology and industrial development capital chain, and create a green technology innovation-industry chain-capital chain with deep integration and efficient synergy of the green technology innovation ecosystem, which, in turn, curbs carbon emissions.

Even though this article has thoroughly addressed the influence of green technology innovation on carbon emissions in the scenario regarding industrial structure upgrading employing econometric techniques, some limitations lie in this study. This article only targets the provincial level administrative areas in China. Future studies can be aimed at the prefectural level, enterprise, or industry level to obtain more precise results. Additionally, resource allocation, economic uncertainty, fiscal decentralization, and education quality are essential components that influence the two associations. Future scholars can focus on the considerations mentioned above to consider the linkage between the two.

## Data availability statement

The original contributions presented in the study are included in the article/supplementary material, further inquiries can be directed to the corresponding authors.

## Author contributions

PG: conceptualization, project administration, writing—review and editing, and writing—original draft. YW: formal analysis, data curation, software, visualization, writing—original draft, and writing—review and editing. XY: methodology, data curation, writing—review and editing, and validation. YZ: writing—review and editing and validation. XS: writing—review and editing, writing—original draft, conceptualization, methodology, funding acquisition, and supervision. XC: writing—review and editing. All authors contributed to the article and approved the submitted version.

## Conflict of interest

The authors declare that the research was conducted in the absence of any commercial or financial relationships that could be construed as a potential conflict of interest.

## Publisher's note

All claims expressed in this article are solely those of the authors and do not necessarily represent those of their affiliated organizations, or those of the publisher, the editors and the reviewers. Any product that may be evaluated in this article, or claim that may be made by its manufacturer, is not guaranteed or endorsed by the publisher.

## Footnotes

^1^See: http://www.stats.gov.cn/xxgk/sjfb/zxfb2020/202102/t20210228_1814159.html.
